# Unraveling the therapeutic mechanisms of Huanglian Jiedu Tang in bacterial meningitis: A comprehensive approach integrating network pharmacology, molecular docking, and experimental validation

**DOI:** 10.1097/MD.0000000000050318

**Published:** 2026-08-14

**Authors:** Yan Xiao, Xiuqing Tong

**Affiliations:** a Neurology Department, Inner Mongolia Medical University Affiliated Hospital, Hohhot, Inner Mongolia Autonomous Region, China.

**Keywords:** bacterial meningitis, Huanglian Jiedu Tang, MAPK14, molecular docking, network pharmacology, *Obacunone*

## Abstract

This study aims to comprehensively elucidate the therapeutic mechanisms of Huanglian Jiedu Tang in bacterial meningitis via an integrated strategy combining network pharmacology, molecular docking, and experimental validation. Network pharmacology was utilized to screen for bioactive constituents, pivotal targets, and associated signaling pathways of *HLJDT*. The potent candidate *Obacunone* and its target MAPK14 were prioritized for verification. An in vitro bacterial meningitis cell model was established, followed by therapeutic interventions using *Obacunone*, the MAPK14 inhibitor VX-702, or their combination. Therapeutic effects were assessed using the CCK-8 assay, sodium ion permeability assays, TUNEL staining, RT-PCR, Western blot, and ELISA. Bioinformatics analysis of the GEO dataset GSE40586 identified 495 BM-related genes, 30 of which intersected with *HLJDT* targets. GO and KEGG enrichment analyses highlighted biological processes including antibiotic and glucocorticoid responses, as well as transcriptional misregulation and HIF-1 signaling pathways. Molecular docking simulations revealed that *Obacunone* possessed superior binding affinity to MAPK14. In the BM cell model, treatment with *Obacunone* or VX-702 alone significantly improved cell viability, attenuated the increased sodium ion permeability, inhibited apoptosis, and suppressed inflammatory cytokine levels. Notably, combination therapy exhibited the most potent synergistic effects. In a bacterial meningitis co-culture model, combined treatment with Obacunone and VX-702, as well as MAPK14 silencing, significantly restored the viability and migratory capacity of HMC3 cells while effectively attenuating early apoptosis. This study delineated the pharmacological underpinnings of *HLJDT* in BM treatment, identifying its active components and targets. Experimental validation confirmed the synergistic action of *Obacunone* and the MAPK14 inhibitor, providing a robust scientific foundation for future drug development and clinical strategies.

## 1. Introduction

Bacterial meningitis (BM), caused by bacterial infection of the meninges, is a life-threatening condition capable of precipitating severe complications, including potentially fatal cerebral infarction in extreme cases.^[1]^ The principal etiological agents of BM include *Streptococcus pneumoniae*, *Neisseria meningitidis*, and *Escherichia coli*.^[2,3]^ Clinically, the disease manifests through a cluster of hallmark symptoms, such as fever, severe headache, nausea, and vomiting.^[4]^

Huanglian Jiedu Tang (*HLJDT*), a traditional Chinese herbal formulation with origins dating back to the Tang Dynasty, consists of *Coptis chinensis*, *Scutellaria baicalensis*, *Phellodendron chinense*, and *Gardenia jasminoides*. This decoction has demonstrated notable efficacy in attenuating neuronal synaptic damage, inhibiting Tau phosphorylation, ameliorating cognitive dysfunction, and alleviating memory deficits induced by microcystin-LR.^[5]^ In neurological disorders, such as ischemic stroke, HLJDT exhibits prophylactic properties. Network pharmacology analyses have identified common signaling pathways, including TNF, HIF-1α, and PI3K-Akt, through which HLJDT modulates neurological disorders.^[6]^ Furthermore, active constituents such as baicalin, wogonoside, and berberine have been shown to mitigate hydrogen peroxide-induced neuronal damage.^[7]^ *HLJDT* also exerts therapeutic effects on diabetic encephalopathy by modulating metabolic pathways, including glycerophospholipid metabolism and fatty acid β-oxidation.^[8]^

Moreover, the formulation attenuates blood–brain barrier (BBB) impairment induced by middle cerebral artery occlusion via the Wnt/β-catenin signaling axis.^[9,10]^ In murine models of high-fat diet-induced obesity, *HLJDT* alleviates cognitive impairment through the TREM2/DAP12/SYK pathway.^[11]^ In Parkinson disease, it confers therapeutic benefits by inhibiting excessive mitochondrial translocation and Pink1 phosphorylation.^[12]^ Regarding inflammatory responses, *HLJDT* suppresses the proliferation of intestinal pathogens, thereby exhibiting anti-inflammatory and antioxidant properties that mitigate ulcerative colitis.^[13]^ Network pharmacology investigations further indicate its efficacy in treating rheumatoid arthritis via IL-17, TNF, and Toll-like receptor (TLR) signaling cascades.^[14]^ Additionally, extracts of the formulation alleviate atopic dermatitis by inhibiting MAPK and NF-κB signaling pathways.^[15]^ In oncology, *HLJDT*, renowned in traditional Chinese medicine (TCM) for its heat-clearing and detoxifying attributes, demonstrates significant potential. Research validates its capacity to inhibit the proliferation and migration of oral squamous cell carcinoma through pleiotropic signaling mechanisms,^[16]^ and to suppress pancreatic cancer progression via the AGE-RAGE and IL-17 signaling pathways.^[17]^

In conclusion, the existing literature underscores the multifaceted efficacy of *HLJDT* in neuroprotection and the management of brain disorders, alongside its therapeutic potential in diverse inflammatory and neoplastic conditions. Employing a network pharmacology approach, this study identifies specific bioactive compounds within *HLJDT*, namely *Kihadalactone A*, *Phellopterin*, *Skimmianin*, *Cavidine*, *Obacunone*, and Phellavin, that contribute to the treatment of BM. Notably, these constituents exhibit favorable molecular docking interactions with MAPK14, a critical marker in meningitis pathology.

## 2. Materials and methods

### 2.1. Comprehensive screening of differentially expressed genes (DEGs) in BM

Microarray data (Gene Expression Omnibus GEO: GSE40586) from 21 BM patients and 18 healthy controls were processed in R. Raw CEL files underwent background correction and normalization using the RMA algorithm, followed by rigorous quality control and batch effect correction. To account for cellular heterogeneity in whole blood, immune cell proportions were estimated via deconvolution and included as covariates. Differential expression analysis was performed using the limma package, defining DEGs with thresholds of |log_2_FC| > 1 and FDR < 0.05.

### 2.2. Systematic identification of bioactive constituents and putative targets

The pharmacologically active constituents of the 4 core botanicals comprising *HLJDT (C chinensis, S baicalensis, P chinense, and G jasminoides*) were retrieved from the TCM Systems Pharmacology Database and Analysis Platform (TCMSP). To ensure the selection of compounds with optimal therapeutic potential, initial screening was conducted based on pharmacokinetic descriptors: oral bioavailability ≥30% and drug-likeness ≥0.18. Additionally, well-documented active compounds reported in the literature that did not meet these strict ADME criteria were also retained for comprehensive analysis. Subsequently, putative and validated target proteins for these ingredients were collected by referencing the TCMSP to mitigate database bias. To standardize nomenclature, all protein names were mapped to official human gene symbols and Entrez IDs using the UniProt Knowledgebase, with custom scripts executing the batch conversion and de-duplication, thereby generating a finalized dataset of gene targets.

### 2.3. Construction of the drug-active ingredient-intersection target network topology

The comprehensive dataset of herbal components, their bioactive ingredients, and the corresponding intersection targets identified in Section 1.2 was imported into Cytoscape 3.7.0 for network visualization and topological analysis. Leveraging the built-in NetworkAnalyzer tool, key topological parameters, including Degree, Betweenness, and Closeness Centrality, were calculated to evaluate the structural importance of specific components and targets within the network. Based on these metrics, nodes were prioritized to construct a comprehensive, visualized “Drug-Active Ingredient-Target” network, thereby elucidating the complex pharmacological interactions and identifying the core therapeutic constituents of the formulation.

### 2.4. Elucidation of the protein–protein interaction (PPI) network architecture

The putative therapeutic targets of *HLJDT* specific to BM pathology were uploaded to the STRING database to comprehensively map functional protein associations. The search parameters are restricted to Homo sapiens, and a confidence score threshold of 0.7 is set to ensure high-confidence interactions. Subsequently, disconnected nodes are removed. The resulting PPI network is imported into Cytoscape 3.7.0 for visualization and topological analysis. To identify the key hub targets regulating the pathological process, the CytoHubba plugin is utilized to rank nodes based on degree and other algorithms, thereby elucidating the core regulatory network.

### 2.5. Gene Ontology (GO) and Kyoto Encyclopedia of Genes and Genomes (KEGG) pathway enrichment analysis

To decipher the biological implications and underlying regulatory mechanisms, the intersecting targets linking *HLJDT* and BM were subjected to comprehensive bioinformatics annotation. GO and KEGG pathway enrichment analyses were performed using the Metascape platform to ensure updated gene annotations. GO analysis characterized gene functions across 3 distinct ontologies: biological process (BP), cellular component (CC), and molecular function (MF). Statistical significance was determined with a stringent threshold of *P* < .01 and FDR (or *q*-value) < 0.05 to minimize false positives. Finally, the prioritized enrichment terms and signaling cascades were visualized using the ChiPlot platform (or specific tool name), thereby elucidating the pharmacological mechanisms.

### 2.6. In silico validation via molecular docking simulation

To further corroborate the binding affinity between the key therapeutic targets and candidate bioactive constituents, a systematic molecular docking analysis was performed. The 3-dimensional crystal structures of the target proteins (*Homo sapiens*) were retrieved from the RCSB Protein Data Bank. Prior to docking, the protein structures were preprocessed by removing water molecules, ions, and original ligands, followed by hydrogen addition and charge optimization to ensure accurate binding site prediction. The small molecule library was curated from the PubChem database. These protein structures and the optimized small molecule compounds were imported into the CB-Dock2 online server, a robust platform for calculating binding modes and affinity. Conformations with the lowest binding energy were prioritized, and docking poses with a Vina score < −7.0 kcal/mol were considered to indicate strong binding activity. Finally, the resultant docking simulations were imported into Discovery Studio for high-resolution visualization and detailed interpretation of molecular interactions.

### 2.7. CCK-8 assay

Human brain microvascular endothelial cells (HBMECs) were seeded into 96-well culture plates at a density of 2000 cells per well. To evaluate cell proliferation kinetics, cell counting kit-8 reagent was introduced into the culture medium at designated time intervals. Subsequently, the plates were incubated at 37°C for 2 hours to facilitate the chromogenic reaction. The absorbance of the resulting solution was quantified at a wavelength of 450 nm using a Varioskan LUX multimode microplate reader to determine cell viability.

### 2.8. Sodium ion permeability assay

To assess the integrity of the endothelial barrier, HBMECs were cultured in Transwell permeable supports at a seeding density of 1*10^5^ cells/mL. Upon achieving approximately 70% confluency, 0.2 mL of sodium fluorescein solution was carefully added to the upper chamber and incubated for 2 hours. The fluorescence intensity of the medium in the lower chamber was subsequently measured using a microplate reader. The permeability of the monolayer to sodium fluorescein was then calculated based on a fluorescence standard curve to quantify paracellular transport.

### 2.9. Western blot analysis

Total cellular proteins were extracted using RIPA lysis buffer supplemented with a protease inhibitor cocktail to prevent degradation. The protein lysates were separated by sodium dodecyl sulfate-polyacrylamide gel electrophoresis and subsequently transferred onto polyvinylidene fluoride membranes. Nonspecific binding sites were blocked by incubating the membranes with 5% skim milk for 1 hour at room temperature. The membranes were then probed overnight at 4°C with specific primary antibodies against MAPK14 (1432-MSM1-P1ABX, Thermo), ZO-1 (61-7300, Thermo), Occludin (ab216327, Abcam), and Claudin-5 (34-1600, Thermo). Following rigorous washing with Tris-buffered saline containing Tween 20, the membranes were incubated with horseradish peroxidase-conjugated secondary antibodies. Specific protein bands were finally visualized using enhanced chemiluminescence reagents.

### 2.10. Reverse transcription polymerase chain reaction (RT-PCR)

Total RNA was isolated using TRIzol reagent, and its purity and concentration were assessed. The RNA was subsequently reverse-transcribed into complementary DNA utilizing the RevertAid First Strand cDNA Synthesis Kit. The cDNA templates were then amplified using FastStart Universal SYBR Premix ExTaq™ II on an ABI PRISM^®^ 7900HT real-time PCR system. Glyceraldehyde-3-phosphate dehydrogenase (GAPDH) served as the endogenous internal control for normalization. Relative mRNA expression levels were quantified using the 2^(-ΔΔCT)^ method. The specific primer sequences utilized for amplification are detailed in Table 1.

**Table 1:** Primer for following genes.

Gene name	Primer sequence
IL-1Β FORWARD	CTGTCCTGCGTGTTGAAAGATG
IL-1Β REVERSE	GGCAGACTCAAATTCCAGCTTG
GAPDH FORWARD	GGAGCGAGATCCCTCCAAAAT
GAPDH REVERSE	GGCTGTTGTCATACTTCTCATGG
TNF-Α FORWARD	CAGGGACCTCTCTCTAATCAGC
TNF-Α REVERSE	GGTTTGCTACAACATGGGCTAC
MAPK14 FORWARD	GCAGGAGCTGAACAAGACAATC
MAPK14 REVERSE	AAAAGCAGCACACACAGAGC
IL-6 FORWARD	AGCCACTCACCTCTTCAGAAC
IL-6 REVERSE	TGCCTCTTTGCTGCTTTCAC

### 2.11. Terminal deoxynucleotidyl transferase dUTP nick end labeling (TUNEL) assay

To evaluate the extent of apoptosis in HBMECs cultured within 6-well plates, a TUNEL assay (Beyotime, C1090) was employed. The cellular specimens were initially fixed with a 4% paraformaldehyde solution for 45 minutes to preserve morphological integrity, followed by permeabilization with 0.3% Triton X-100 for 20 minutes to facilitate intracellular access. Subsequently, the cells were incubated with the TUNEL reaction mixture at 37°C for 45 minutes to enzymatically label DNA strand breaks. For nuclear visualization, the specimens were stained with DAPI for 5 minutes. Finally, fluorescence imaging was performed using a fluorescence microscope (Nikon Eclipse Ti2, Tokyo, Japan) to capture and document the apoptotic events.

### 2.12. Enzyme-linked immunosorbent assay (ELISA)

The concentrations of specific inflammatory mediators were determined using a standard ELISA protocol. Initially, 96-well plates were coated with capture antibodies and incubated overnight at 4°C to ensure optimal antigen binding. Following a rigorous washing step to remove unbound antibodies, 100 µL of cell culture supernatant was aliquoted into the wells and incubated at 37°C for 2 hours. After subsequent washing, 100 µL of a biotinylated detection antibody was introduced and incubated at 37°C for 1 hour to facilitate complex formation. The plates were then washed again, and 100 µL of horseradish peroxidase-conjugated streptavidin was added, followed by a 30-minute incubation at 37°C. To initiate the colorimetric reaction, 100 µL of TMB substrate was added and incubated in the dark at 25°C for 15 minutes. The enzymatic reaction was terminated by the addition of 2 M sulfuric acid, and the absorbance was measured at 450 nm using a microplate reader. The concentrations of the target cytokines, specifically TNF-α, IL-1β, and IL-6, were quantified by interpolation from standard curves.

### 2.13. Statistical analysis

All quantitative data are expressed as the mean ± standard deviation (SD). Each experiment was performed in triplicate. Statistical analyses were conducted using GraphPad Prism 8.0 software (GraphPad Software, LLC). Comparisons among multiple groups were analyzed using 1-way analysis of variance (ANOVA) followed by Tukey’s post hoc test to identify specific differences. For comparisons between 2 groups, an unpaired Student *t* test was utilized. A *P*-value of <.05 was considered to indicate statistical significance.

## 3. Results

### 3.1. Identification of BM disease targets

**Figure 1.**
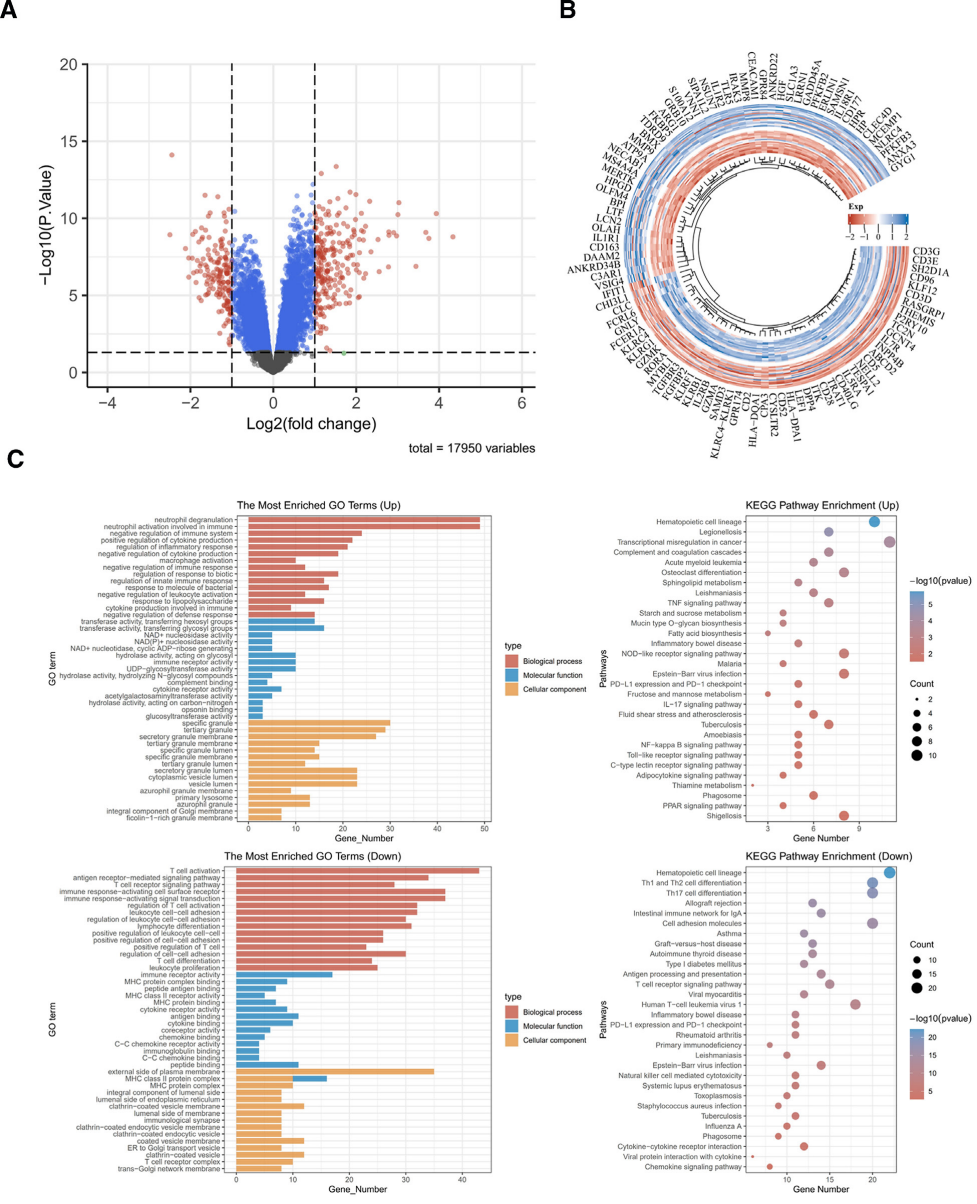
Identification of disease targets associated with BM. (A) A volcano plot depicts the comparative transcriptomic RNA analysis between BM patients and healthy controls, identifying 235 significantly upregulated genes and 165 significantly downregulated genes. (B) A circular heatmap visualizes the hierarchical expression profiles and differential clustering of upregulated and downregulated genes. (C) GO and KEGG elucidate the key biological processes, molecular functions, cellular components, and signaling pathways mechanistically associated with the DEGs in BM. The primary source data are retrievable from “Original data-1”. BM = bacterial meningitis, DEG = differentially expressed gene, GO = Gene Ontology, KEGG = Kyoto Encyclopedia of Genes and Genomes.

Comparative RNA analysis of BM patients versus healthy controls identified differential expression in 17,950 genes. Among these, 235 genes were significantly upregulated, while 165 were downregulated (Fig. 1A).

A circular heatmap highlighted the top ten upregulated genes: *CD3G*, *CD3E*, *SH2D1A*, *CD96*, *KLF12*, *CD3D*, *RASGRP1*, *THEMIS*, and *P2RY10*. Conversely, the top ten downregulated genes included *GYG1*, *ANXA3*, *PFKFB3*, *NLRC4*, *MCEMP1*, *CLEC4D*, *HP*, *HPR*, *CD177*, and *IL18R1* (Fig. 1B).

GO functional enrichment analysis revealed the top 3 upregulated biological processes: neutrophil degranulation, neutrophil activation involved in immune response, and negative regulation of the immune system. Key molecular functions included transferase activity (transferring hexosyl groups), transferase activity (transferring glycosyl groups), and NAD+ nucleosidase activity. The predominant cellular components were specific granule, tertiary granule, and secretory granule membrane. For downregulated genes, the top biological processes were T cell activation, antigen receptor-mediated signaling pathway, and T cell receptor signaling pathway. The leading molecular functions were immune receptor activity, MHC protein complex binding, and peptide antigen binding. The primary cellular components included the external side of the plasma membrane, MHC class II protein complex, and MHC protein complex.

KEGG pathway enrichment analysis identified the top 5 upregulated pathways: hematopoietic cell lineage, legionellosis, transcriptional misregulation in cancer, complement and coagulation cascades, and acute myeloid leukemia. The top 5 downregulated pathways were hematopoietic cell lineage, Th1 and Th2 cell differentiation, Th17 cell differentiation, allograft rejection, and intestinal immune network for IgA (Fig. 1C).

### 3.2. Analysis of intersection targets between BM and HLJDT

**Figure 2.**
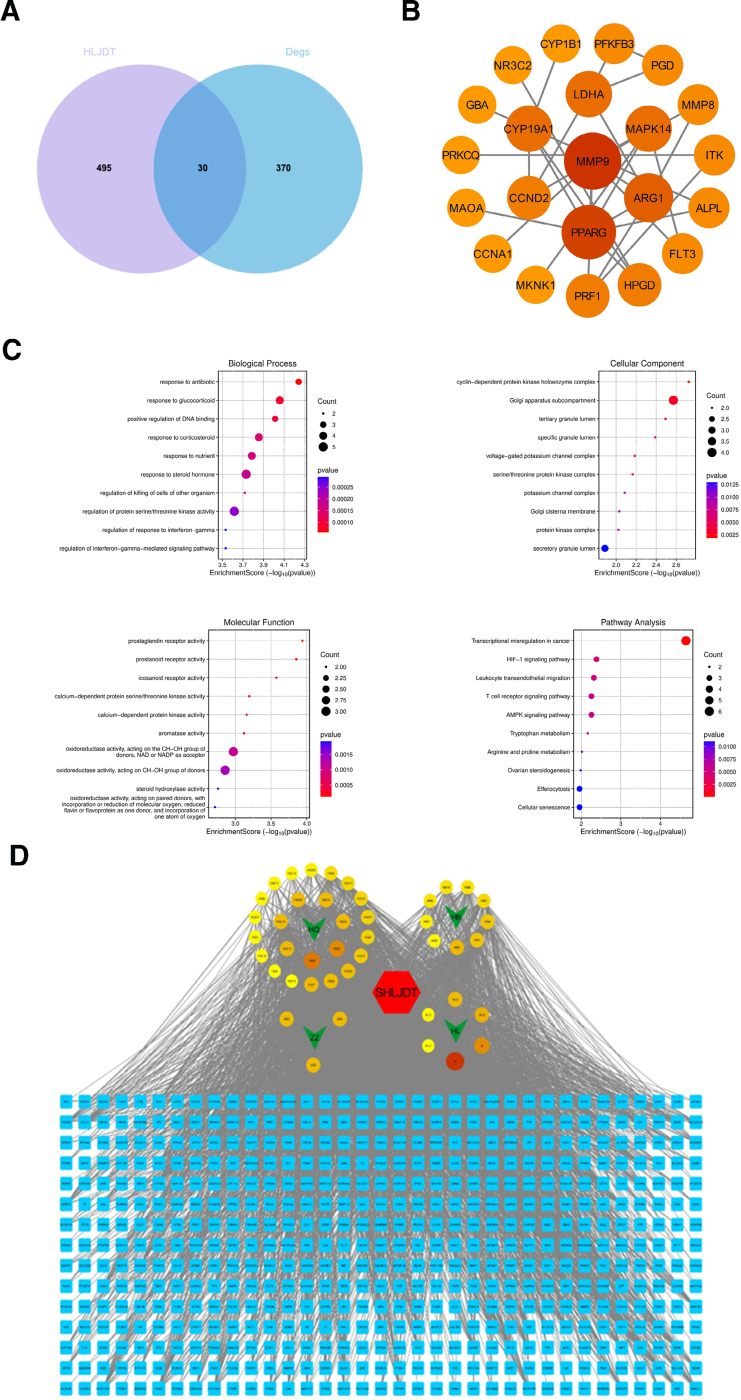
Integrative analysis of intersection targets between BM and *HLJDT*. (A) A Venn diagram highlights the overlapping genes identified from the GSE40586 dataset and the target genes of *HLJDT*. (B) A PPI network, constructed using the STRING database and analyzed with Cytoscape 3.7.0, identifies the top target proteins based on confidence scores. (C) Enrichment analysis of the intersection targets uncovers the primary biological processes, molecular functions, cellular components, and KEGG pathways involved. (D) A target-molecule network diagram delineates the complex pharmacological relationships between the active components of Huanglian Jiedu Tang and their corresponding target genes in the context of BM. The primary source data are retrievable from “Original data-1”. BM = bacterial meningitis, HLJDT = Huanglian Jiedu Tang, KEGG = Kyoto Encyclopedia of Genes and Genomes, PPI = protein–protein interaction.

From the GSE40586 dataset in the GEO database, 495 BM-related genes were identified. Potential targets of *HLJDT* were analyzed using the TCMSP database, yielding 30 common genes as potential therapeutic targets for BM (Fig. 2A).

These common targets were further analyzed using the STRING database to construct a PPI network. Topological analysis via Cytoscape 3.7.0 identified the top proteins: *NR3C2*, *GBA*, *PRKCQ*, *MAOA*, *CCNA1*, *MKNK1*, *CYP1B1*, *PFKFB3*, *PGD*, *MMP8*, *ITK*, *ALPL*, *FLT3*, *HPGD*, *PRF1*, PPARG, *MMP9*, *ARG1*, MAPK14, *LDHA*, *CYP19A1*, and *CCND2. MMP9* and MAPK14 emerged as the most central genes (Fig. 2B).

Enrichment analysis of the intersection targets revealed the top 5 biological processes: response to antibiotics, response to glucocorticoids, positive regulation of DNA binding, response to corticosteroids, and response to nutrients. The top molecular functions were prostaglandin receptor activity, prostanoid receptor activity, icosanoid receptor activity, calcium-dependent protein serine/threonine kinase activity, and calcium-dependent protein kinase activity. The primary cellular components included the cyclin-dependent protein kinase holoenzyme complex, Golgi apparatus subcompartment, tertiary granule lumen, specific granule lumen, and voltage-gated potassium channel complex. The top KEGG pathways were transcriptional misregulation in cancer, HIF-1 signaling pathway, leukocyte transendothelial migration, T cell receptor signaling pathway, and AMPK signaling pathway (Fig. 2C).

A network diagram was constructed using Cytoscape 3.7 to visualize the relationships between the active components of *HLJDT* and their corresponding target genes in BM (Fig. 2D).

### 3.3. HUB gene screening and protein interaction mapping

**Figure 3.**
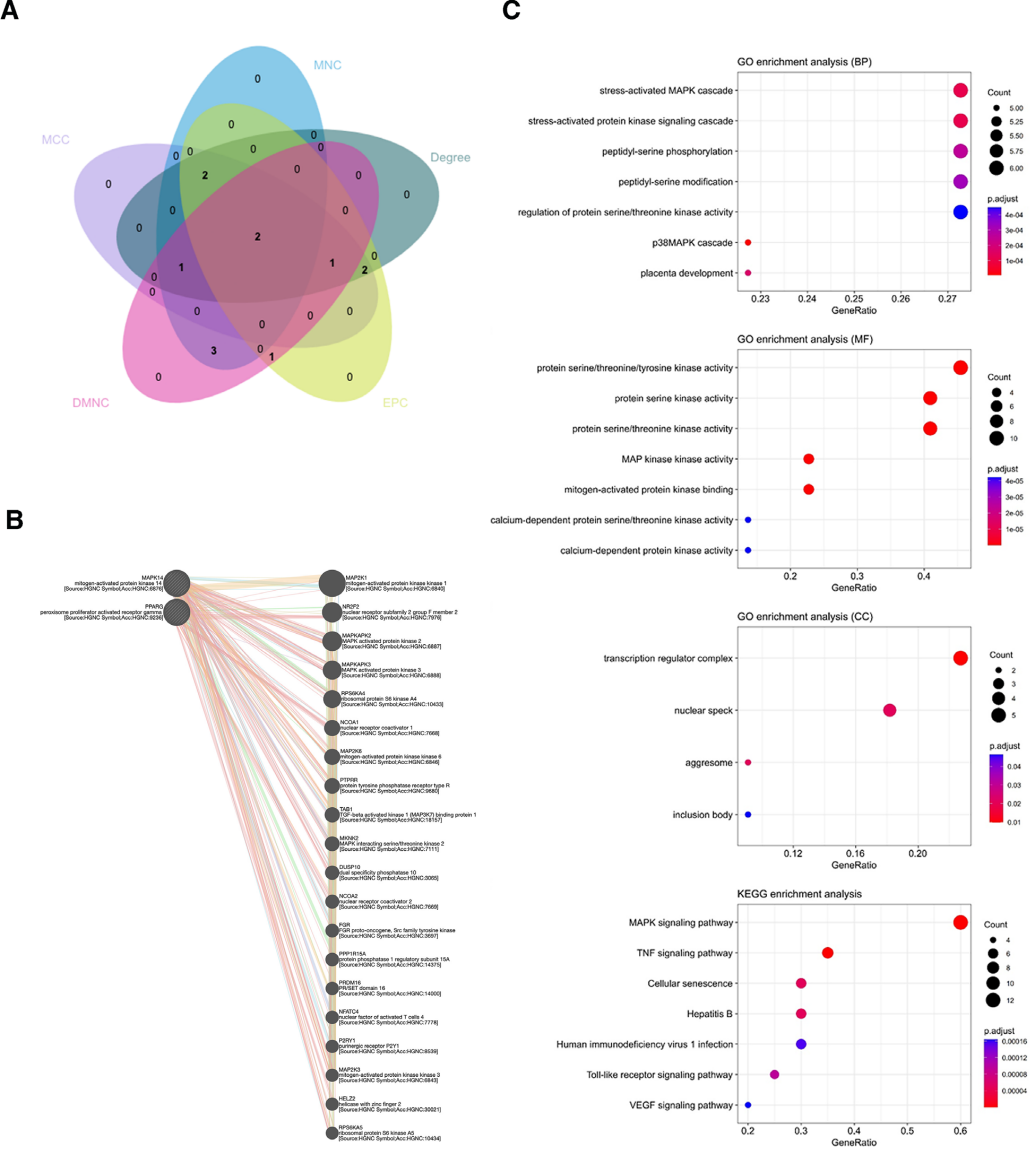
Hub gene screening and protein interaction mapping. (A) Topological analysis conducted with Cytoscape 3.7 utilizing the CytoHubba plugin identifies PPARG and MAPK14 as the 2 most prominent hub genes. (B) Network topology analysis elucidates the intricate interactions among PPARG, MAPK14, and associated proteins, encompassing physical associations, co-expression patterns, and pathway linkages. (C) GO and KEGG analysis of the 20 proteins interacting with PPARG and MAPK14 underscores the predominant biological processes, molecular functions, cellular components, and signaling pathways engaged. The primary source data are retrievable from “Original data-1”. GO = Gene Ontology, KEGG = Kyoto Encyclopedia of Genes and Genomes.

Topological analysis using Cytoscape 3.7 with algorithms such as Maximal Clique Centrality, Maximum Neighborhood Component, Degree, Edge Percolated Component, and Density of Maximum Neighborhood Component identified 2 hub genes: PPARG and MAPK14 (Fig. 3A).

Network topology analysis revealed interactions between PPARG, MAPK14, and interacting proteins such as MAP2K1, NR2F2, MAPKAPK2, MAPKAPK3, RPS6KA4, NCOA1, MAP2K6, PTPRR, TAB1, MKNK2, DUSP10, NCOA2, FGR, PPP1R16A, PRDM16, NFATC4, P2RY1, MAP2K3, HELZ2, and RPS6KA5. These interactions included physical interactions, co-expression, predicted interactions, co-localization, genetic interactions, pathway associations, and shared protein domains (Fig. 3B).

GO and KEGG analysis of the 20 proteins interacting with PPARG and MAPK14 revealed the top biological processes: stress-activated MAPK cascade, stress-activated protein kinase signaling cascade, and peptidyl-serine phosphorylation. The top molecular functions were protein serine/threonine/tyrosine kinase activity, protein serine kinase activity, and protein serine/threonine kinase activity. The primary cellular components were the transcription regulator complex, nuclear speck, and aggresome. The top signaling pathways were the MAPK signaling pathway, TNF signaling pathway, and cellular senescence (Fig. 3C).

### 3.4. Molecular docking experiments

**Figure 4.**
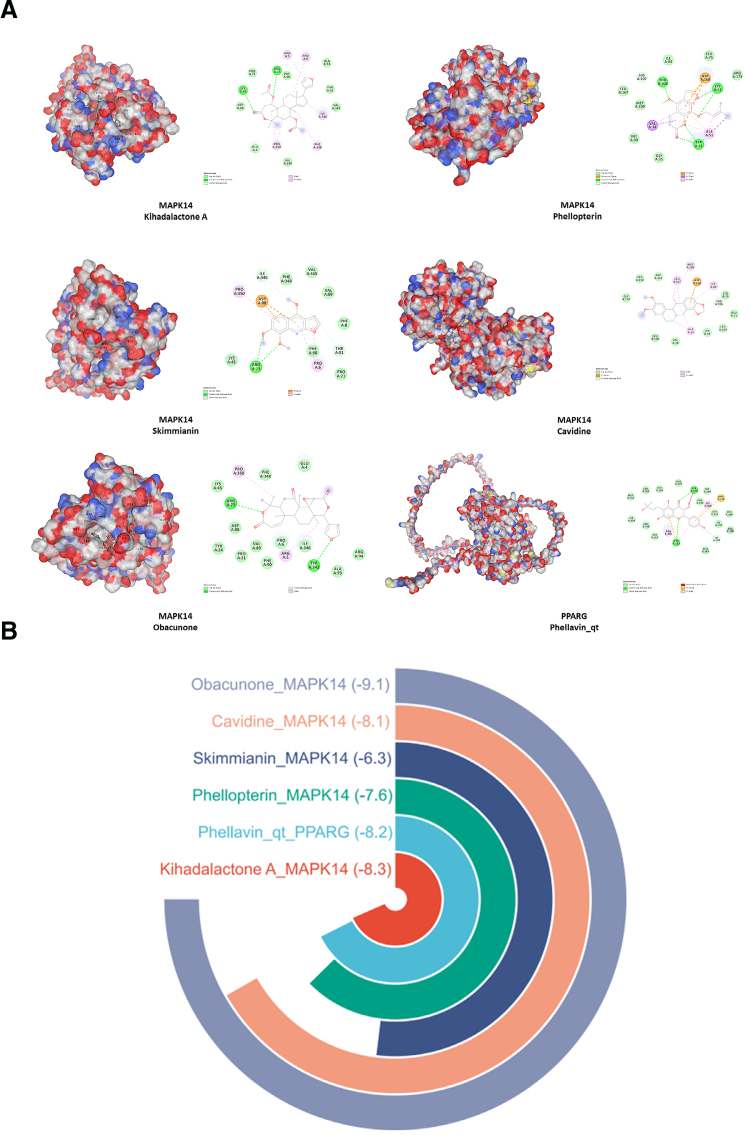
Molecular docking simulations and validation. (A) Molecular docking experiments demonstrate the binding affinity of core compounds from *HLJDT* (Kihadalactone A, Phellopterin, Skimmianin, Cavidine, *Obacunone*, Phellavin_qt) to MAPK14, and Phellavin_qt to PPARG. (B) A circular results diagram ranks the inhibitory effects of the compounds based on binding energy, with the *Obacunone*-MAPK14 complex exhibiting the most potent inhibitory activity. The primary source data are retrievable from “Original data-1”. HLJDT = Huanglian Jiedu Tang.

Core compounds of *HLJDT*, such as Kihadalactone A, Phellopterin, Skimmianin, Cavidine, *Obacunone*, and Phellavin (derived from *P chinense*), were docked with MAPK14. Phellavin was also docked with PPARG. Binding energies were all negative, with *Obacunone* showing the strongest affinity for MAPK14 (Fig. 4A).

A circular plot indicated that, based on binding energy, *Obacunone*_MAPK14 had the highest binding affinity, followed by Cavidine_MAPK14, Kihadalactone_A_MAPK14, and Phellopterin_MAPK14, while Skimmianin_MAPK14 had the weakest effect (Fig. 4B).

### 3.5. Synergistic therapeutic effects of HLJDT monomer Obacunone and MAPK14 inhibitor in BM

To validate the therapeutic effects of *HLJDT* in BM, a cell model was established by co-culturing 1*10^7^ *E coli* in the upper chamber of a Transwell system with HBMECs (1 × 10^5^ cells) in the lower chamber for 24 hours. Inactivated *E coli* served as the negative control. Treatments included *Obacunone*, the MAPK14 inhibitor VX-702, and their combination. To ascertain the optimal dosages of Obacunone and VX-702, their dose- and time-dependent effects were evaluated via CCK-8 assay. Data indicated that under the bone metastasis model, the effective concentrations were established at 20 μM for Obacunone and 1 μM for VX-702 (Fig. S1, Supplemental Digital Content 1).

**Figure 5.**
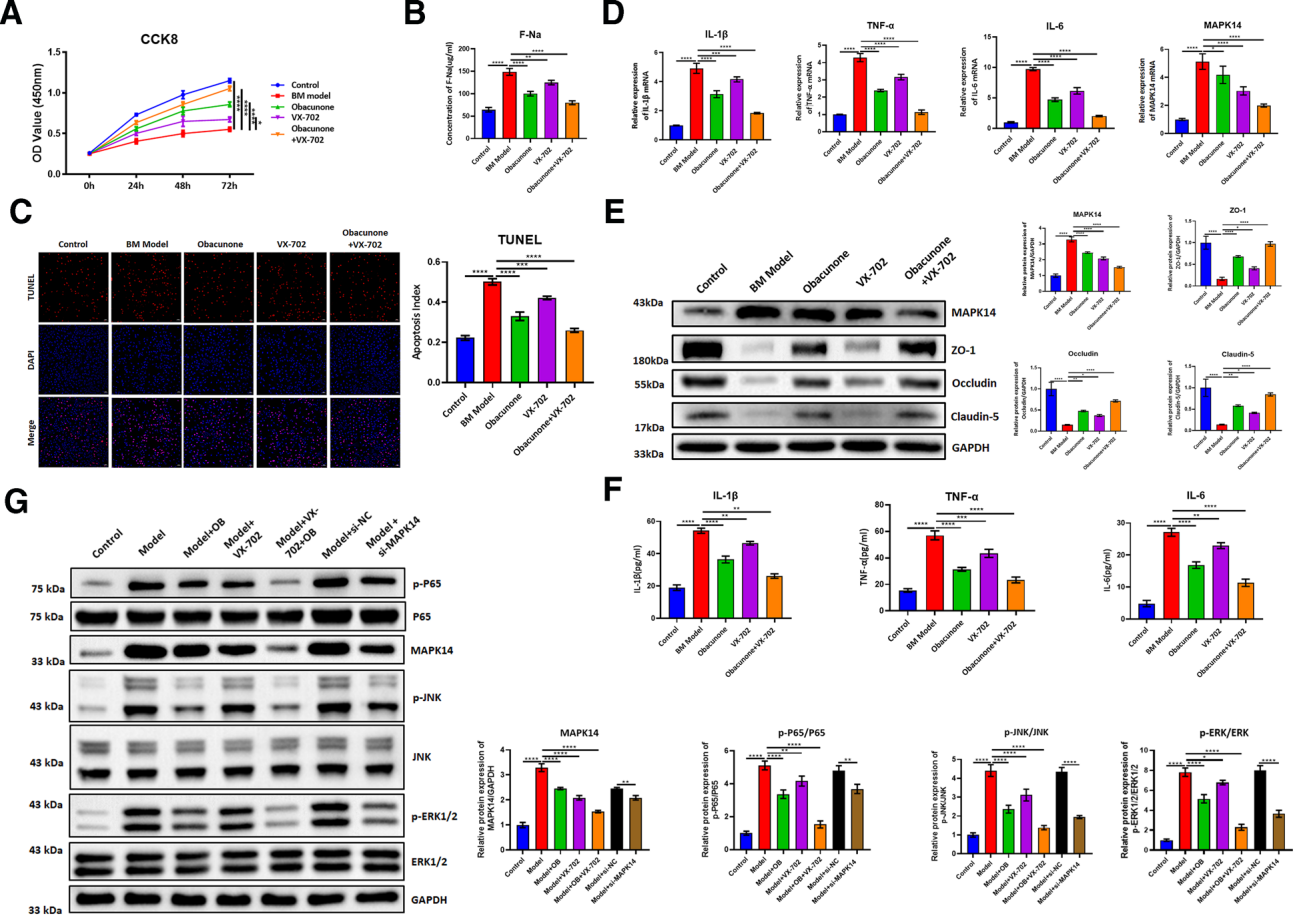
Synergistic therapeutic effects of the *HLJDT* monomer *Obacunone* and MAPK14 inhibitor in BM. (A) CCK-8 assay results depict cell viability across the control group, BM model group, *Obacunone*-treated group, VX-702-treated group, and the combined *Obacunone*+VX-702-treated group. The data are presented as mean ± SD. Data were evaluated using the 2-way ANOVA followed by Tukey’s multiple comparisons test. The cell experiments were independently repeated 3 times. (B) Sodium ion concentration assays illustrate intracellular sodium ion levels in the control group, BM model group, *Obacunone*-treated group, VX-702-treated group, and the combined *Obacunone*+VX-702-treated group. The data are presented as mean ± SD. Data were evaluated using the 1-way ANOVA followed by Tukey’s multiple comparisons test. The cell experiments were independently repeated 3 times. (C) TUNEL assays quantify the apoptosis rate in the control group, BM model group, *Obacunone*-treated group, VX-702-treated group, and the combined *Obacunone*+VX-702-treated group. The data are presented as mean ± SD. Data were evaluated using the 1-way ANOVA followed by Tukey’s multiple comparisons test. The cell experiments were independently repeated 3 times. The primary source data are retrievable from “Original data-1.” (D) RT-PCR results reveal the relative mRNA expression levels of IL-1β, TNF-α, IL-6, and MAPK14 in the control group, BM model group, *Obacunone*-treated group, VX-702-treated group, and the combined *Obacunone*+VX-702-treated group. The data are presented as mean ± SD. Data were evaluated using the 1-way ANOVA followed by Tukey’s multiple comparisons test. The cell experiments were independently repeated 3 times. (E) Western blot analysis demonstrates the protein expression levels of MAPK14, ZO-1, Occludin, and Claudin-5 in the control group, BM model group, *Obacunone*-treated group, VX-702-treated group, and the combined *Obacunone*+VX-702-treated group. The data are presented as mean ± SD. Data were evaluated using the 1-way ANOVA followed by Tukey’s multiple comparisons test. The cell experiments were independently repeated 3 times. (F) ELISA results indicate the secretion levels of inflammatory factors IL-1β, TNF-α, and IL-6 in the control group, BM model group, *Obacunone*-treated group, VX-702-treated group, and the combined *Obacunone*+VX-702-treated group. The data are presented as mean ± SD. Data were evaluated using the 1-way ANOVA followed by Tukey’s multiple comparisons test. The cell experiments were independently repeated 3 times. (G) The protein expression levels of MAPK14, p-P65/P65, p-JNK/JNK, and p-ERK/ERK were determined by Western blot analysis in the control group, BM model group, *Obacunone*-treated group, VX-702-treated group, *Obacunone*+VX-702-treated group, BM model+si-NC group, and BM model+si-MAPK14 group. Data are presented as mean ± SD. Statistical analysis was performed using 1-way ANOVA followed by Tukey’s post hoc multiple comparisons test. All cell experiments were independently repeated 3 times. The primary source data are retrievable from “Original data-2”. BM = bacterial meningitis, CCK-8 = cell counting kit-8, OD = optical density, RT-PCR = reverse transcription polymerase chain reaction, SD = standard deviation.

Cell viability was significantly reduced in the BM model group compared to the control. Treatment with *Obacunone* or VX-702 alone increased cell viability, whereas the combination treatment resulted in the most significant improvement (Fig. 5A).

Sodium ion concentration was significantly higher in the BM model group than in the control. Treatment with *Obacunone* or VX-702 alone reduced sodium ion concentration, while the combination treatment showed the most significant reduction (Fig. 5B).

The BM model group exhibited a significantly higher apoptosis rate compared to the control. Treatment with *Obacunone* or VX-702 alone slightly reduced apoptosis, while the combination treatment resulted in the most significant reduction (Fig. 5C).

Compared to the control, the BM model group showed significantly elevated levels of IL-1β, TNF-α, IL-6, and MAPK14 mRNA. Treatment with *Obacunone* or VX-702 alone reduced these levels, with the combination treatment showing the most significant reduction (Fig. 5D).

Compared with the control, the BM model group displayed elevated MAPK14 protein levels and reduced levels of ZO-1, occludin, and claudin-5. Treatment with *Obacunone* or VX-702 alone slightly decreased MAPK14 levels and increased tight junction protein levels; notably, the combination treatment induced the most significant changes (Fig. 5E).

Compared with the control, the BM model group demonstrated elevated levels of IL-1β, TNF-α, and IL-6. Treatment with *Obacunone* or VX-702 alone slightly reduced these levels, while the combination treatment achieved the most significant reduction (Fig. 5F).

To elucidate the regulatory impact of *Obacunone*, VX-702, or MAPK14 silencing on the p-P65/P65, p-JNK/JNK, and p-ERK/ERK signaling cascades within co-cultured HBMECs, Western blot analysis was employed. The findings demonstrated a marked upregulation in the protein abundance of MAPK14, p-P65/P65, p-JNK/JNK, and p-ERK/ERK in the Model group relative to the Control group. Relative to the Model group, individual treatment with *Obacunone* or VX-702 elicited a modest attenuation in the expression of these proteins; notably, this suppressive effect was significantly potentiated following the combined administration of *Obacunone* and VX-702. Moreover, the protein expression levels of MAPK14, p-P65/P65, p-JNK/JNK, and p-ERK/ERK were observed to be distinctly diminished in the Model+si-MAPK14 group compared to the Model+si-NC group (Fig. 5G).

### 3.6. Evaluation of Obacunone, VX-702, and MAPK14 silencing effects on HMC3 cells in a bacterial meningitis co-culture model

**Figure 6.**
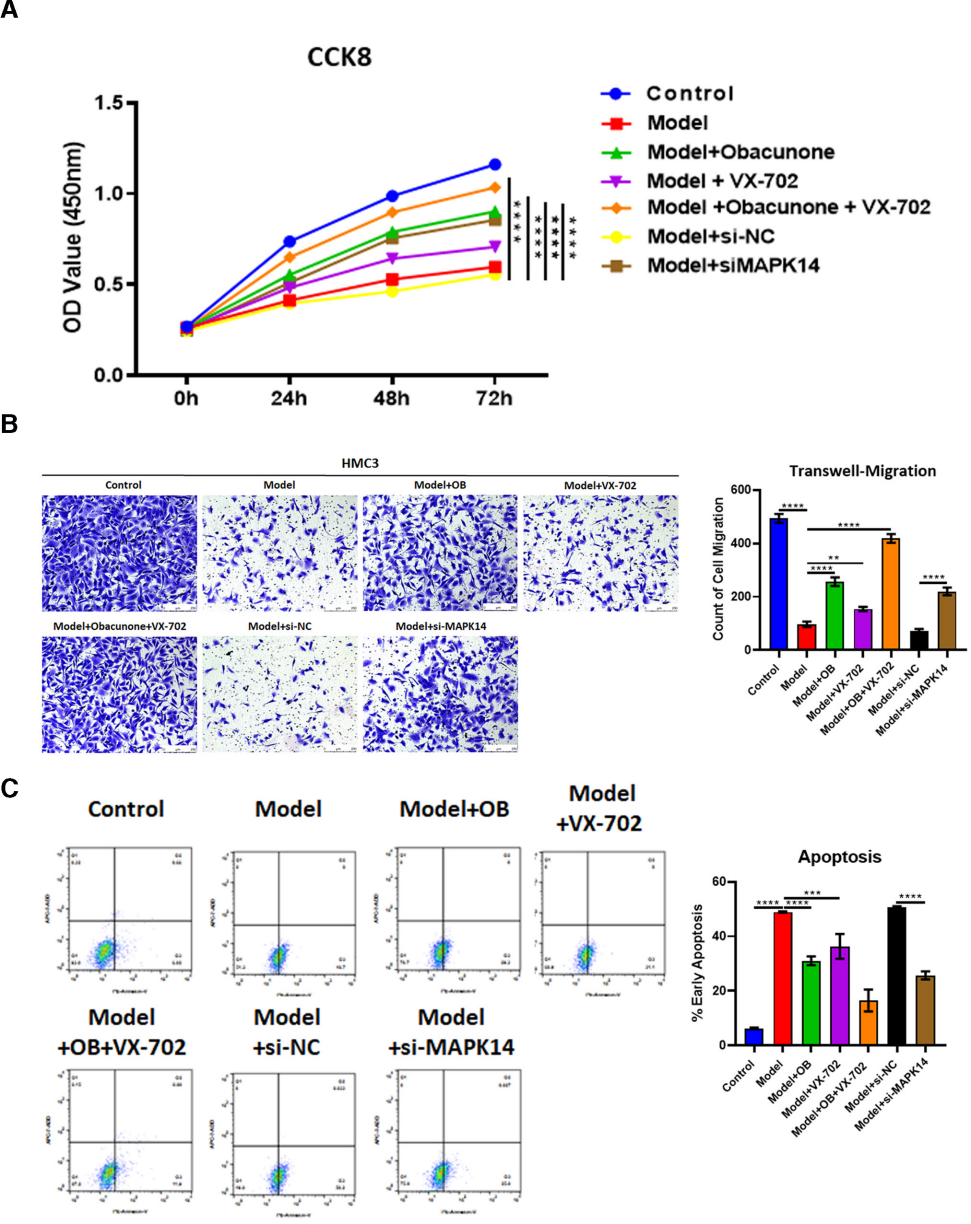
Evaluation of *Obacunone*, VX-702, and MAPK14 silencing effects on HMC3 cells in a bacterial meningitis co-culture model. (A) The proliferative capacity of human microglial HMC3 cells across the Control, Model, Model+*Obacunone*, Model+VX-702, Model+*Obacunone*+VX-702, Model+si-NC, and Model+si-MAPK14 cohorts was quantitatively assessed by measuring the optical density (OD) at 450 nm at 24, 48, and 72 hours posttreatment utilizing the CCK-8 assay. All acquired data are expressed as the mean ± SD. Statistical significance was determined via 1-way ANOVA coupled with Tukey’s post hoc test for multiple comparisons. To ensure robustness and reproducibility, all cellular experiments were conducted in triplicate with 3 independent biological replicates. (B) The migratory capability of human microglial HMC3 cells within the Control, Model, Model+*Obacunone*, Model+VX-702, Model+*Obacunone*+VX-702, Model+si-NC, and Model+si-MAPK14 groups was rigorously evaluated employing the Transwell migration assay. Experimental data are reported as the mean ± SD. Statistical comparisons were performed using 1-way analysis of ANOVA followed by Tukey’s post hoc multiple comparisons test. The validity of the findings was confirmed by repeating all cell experiments independently 3 times. The primary source data are retrievable from “Original data-3.” (C) The extent of early apoptosis in human microglial HMC3 cells belonging to the Control, Model, Model+*Obacunone*, Model+VX-702, Model+*Obacunone*+VX-702, Model+si-NC, and Model+si-MAPK14 cohorts was precisely determined through flow cytometric analysis. Data are articulated as mean ± SD. Statistical evaluation was carried out using 1-way ANOVA in conjunction with Tukey’s post hoc multiple comparisons test. Each experimental procedure was independently replicated 3 times to ensure reliability. The primary source data are retrievable from “Original data-3”. BM = bacterial meningitis, CCK-8 = cell counting kit-8, OD = optical density, SD = standard deviation.

To faithfully recapitulate the intricate pathological progression of bacterial meningitis within the brain microenvironment, human microglial HMC3 cells were subjected to stimulation with the co-culture conditioned medium for a duration of 48 hours. Subsequently, cell viability was rigorously quantified using the CCK-8 assay. The experimental data indicated that the viability of HMC3 cells in the Model group was significantly compromised compared to the Control group. Relative to the Model group, the viability of HMC3 cells treated with *Obacunone* or VX-702 alone exhibited a modest restoration; however, the combined administration of *Obacunone* and VX-702 elicited a substantially more pronounced enhancement in cell viability. Furthermore, the cell viability in the Model+si-MAPK14 group was significantly elevated compared to that observed in the Model+si-NC group (Fig. 6A).

To mimic the pathological trajectory of intracranial bacterial meningitis, HMC3 cells were exposed to the co-culture conditioned medium for 48 hours, followed by a comprehensive evaluation of migratory capacity utilizing the Transwell assay. The results demonstrated that the migration ratio of HMC3 cells in the Model group was significantly suppressed relative to the Control group. In contrast to the Model group, the migration ratio of HMC3 cells treated with *Obacunone* or VX-702 alone displayed a marginal increase; notably, combined treatment with *Obacunone* and VX-702 led to a significant augmentation in the migration ratio. Additionally, the migration ratio in the Model+si-MAPK14 group was markedly increased compared to the Model+si-NC group (Fig. 6B).

To simulate the pathophysiological milieu of bacterial meningitis in the brain, HMC3 cells were stimulated with the co-culture conditioned medium for 48 hours, and the extent of early apoptosis was precisely determined via flow cytometry. The findings revealed that the proportion of early apoptotic HMC3 cells in the Model group was significantly elevated compared to the Control group. When compared with the Model group, the early apoptosis ratio of HMC3 cells treated with *Obacunone* or VX-702 alone was slightly attenuated, whereas the combination of *Obacunone* and VX-702 resulted in a significantly diminished early apoptosis ratio. Moreover, the early apoptosis ratio in the Model+si-MAPK14 group was significantly lower than that in the Model+si-NC group (Fig. 6C).

## 4. Discussion

Meningitis is principally stratified into bacterial and viral etiologies.^[18]^ BM represents a critical and life-threatening infectious pathology. Epidemiological surveillance over the preceding quinquennium indicates that pediatric patients account for 82% of the total cases. Furthermore, this demographic is notably susceptible to severe sequelae, including postinfectious hydrocephalus and seizure disorders.^[19]^ The characteristic clinical manifestations encompass pyrexia, nuchal rigidity, and headache. Standard therapeutic protocols predominantly necessitate antimicrobial regimens, frequently augmented with adjuvant glucocorticoids and osmotic agents such as mannitol.^[20,21]^

TCM possesses distinctive efficacy in the domain of antibacterial therapies. The sophisticated molecular mechanisms underpinning its antimicrobial effects are predominantly characterized by the disruption of bacterial membrane permeability, the biosynthetic suppression of essential proteins and nucleic acids, and the functional modulation of critical enzymatic activities.^[22]^ *HLJDT,* a renowned classical formulation within the TCM pharmacopeia, is rich in diverse bioactive constituents that orchestrate multifaceted pharmacological mechanisms. These encompass the potent attenuation of inflammatory cascades, the restoration of immune homeostasis, the modulation of vascular dysfunction, the inhibition of oncogenic processes, and the significant amelioration of cerebral ischemia-reperfusion injury.^[23]^ *HLJDT* exerts a potent inhibitory effect on the bacterial load within infected tissues while concomitantly attenuating the levels of local inflammatory mediators. Mechanistically, through the downregulation of protein expression levels associated with the TLR2/MyD88/NF-κBp65 signaling axis, this formulation effectively mediates the suppression of the systemic inflammatory response.^[24]^ In the context of neurological pathologies, *HLJDT* demonstrates the capacity to ameliorate gut dysbiosis, mitigate the pathological accumulation of gut-microbiota-associated amyloid-β, and profoundly alleviate neuroinflammatory cascades.^[25]^ Furthermore, with respect to intestinal pathology, the decoction attenuates the progression of inflammation by significantly reducing pro-inflammatory cytokine levels and myeloperoxidase activity within the colon, a function primarily mediated through the targeted modulation and inhibition of the JAK2/STAT3 transduction pathway.^[26]^

*HLJDT* orchestrates its therapeutic efficacy by modulating the HIF-1α/MMP9 signaling axis via the intermediary action of the pivotal biomarker succinate.^[27]^ The synergistic combination of *C chinensis* and *Zingiber officinale* exerts a profound suppressive effect on glucose metabolism within gastric cancer cells, thereby manifesting antitumor activity through the marked downregulation of LDHA gene expression.^[28]^ Regarding its antipyretic properties, *HLJDT* functions to attenuate the release of inflammatory and pyrogenic mediators through the inhibition of the MAPK signaling cascade, a mechanism evidenced by the decreased protein expression of p-ERK, p-JNK, and p-P38.^[29]^ Furthermore, *HLJDT* regulates the inflammatory tumor microenvironment and impedes breast cancer progression by targeting tumor immunosuppression; specifically, it downregulates ARG1 levels via the inhibition of the RhoA/ROCK transduction pathway.^[30]^ Finally, *HLJDT* confers hepatoprotective effects against drug-induced liver injury via the tryptophan metabolic pathway, functioning through the modulation of the genes *CYP1A1*, *CYP1A2*, and *CYP1B1*.^[31]^ In the scope of the present investigation, the STRING database was used to construct a PPI network based on intersecting targets. This network was then subjected to topological analysis using Cytoscape 3.7.0 software. Through this systematic evaluation, a set of pivotal core targets was identified, namely PPARG, *ARG1*, MAPK14, *LDHA*, *CYP19A1*, and *CCND2*. Notably, these entities occupy a central position within the overarching regulatory architecture. This pronounced topological prominence implies their probable function as critical biomarkers or key effector molecules that mediate the therapeutic efficacy of the formulation. Consequently, these findings provide promising candidate targets, laying a vital theoretical foundation for subsequent in-depth investigations into the underlying molecular mechanisms.

Within the clinical management of BM, the expeditious administration of optimized antibiotic regimens frequently precipitates therapeutic outcomes characterized by sustained defervescence over 48 hours and the complete resolution of meningeal irritation signs.^[32]^ Furthermore, glucocorticoids serve as potent pharmacological agents, demonstrating exemplary efficacy in achieving clinical remission among patients presenting with anti-MOG antibody-positive pathology. Notably, these subjects exhibited successful convalescence to corticosteroid intervention, which was necessitated by the lack of response to antibiotic therapies initially prescribed for suspected bacterial etiologies.^[33]^ In the context of acute BM, the adjunctive utilization of these agents is correlated with a statistically significant reduction in mortality rates, alongside the effective attenuation of deleterious inflammatory cascades and the comprehensive alleviation of clinical symptom phenotypes.^[34]^ Beyond these therapeutic considerations, transcriptional dysregulation is firmly established as a pivotal upstream mechanism propelling the pathogenesis of rheumatoid arthritis and senescence-associated inflammation, thereby playing a contributory role in the propagation of the inflammatory milieu.^[35]^

At the molecular level, hypoxia-inducible factor-1 functions as a critical molecular switch that mechanistically links infectious processes to the onset of meningitis. Specifically, infection by *Pasteurella multocida* induces the robust activation of HIF-1α, which subsequently downregulates structural proteins essential for the maintenance of BBB integrity. The loss of these proteins results in increased BBB permeability or “leakage,” thereby facilitating bacterial translocation into the central nervous system and precipitating meningitic pathology.^[36]^ Concurrently, leukocyte transendothelial migration represents the primary route via which Enterovirus A71 invades the CNS to induce meningitis; this phenomenon is increasingly understood not merely as a defensive immunological reaction, but as a mechanism exploited by EV-A71 to breach the BBB.^[37]^ Furthermore, clinical observations indicate that the onset of meningitis is accompanied by a downregulation of FYN expression and a concomitant reduction in the proportion of Natural Killer T cells, suggesting that T-cell receptor-dependent protective mechanisms are significantly compromised during the progression of the disease.^[38]^

In the context of the present investigation, GO enrichment analysis elucidated that biological processes are predominantly implicated in responses to antibiotic and hormonal stimuli, thereby suggesting perturbations in drug metabolic processes and inflammatory regulatory mechanisms. The enrichment of cellular components pointed to aberrations associated with secretory pathways and cell cycle structures, whereas molecular function analysis accentuated the pivotal roles of prostaglandin receptor activity and calcium ion transport. Additionally, KEGG pathway analysis delineated a sophisticated regulatory network encompassing inflammation, immunity, and metabolism. The prominence of transcriptional dysregulation suggests a foundational disequilibrium in gene expression profiles. Moreover, the activation of the HIF-1 signaling pathway was found to be intricately associated with leukocyte transendothelial migration, shedding light on the mechanisms underlying immune cell infiltration. The identification of the T-cell receptor signaling pathway corroborates the involvement of adaptive immunity, while the AMPK signaling pathway highlights the critical importance of cellular energy metabolic reprogramming. Collectively, these findings indicate that the pathological trajectory involves a complex, multi-dimensional network comprising drug responsiveness, inflammatory mediator signaling, immune cell migration, and metabolic adaptation, thus providing a robust theoretical framework for future mechanistic investigations and the strategic screening of potential therapeutic targets.

Capitalizing on the distinctive anti-inflammatory and antibacterial efficacy of *HLJDT*, we systematically screened the TCMSP database to elucidate its bioactive constituents, based on the formulation ratio of 3:2:2:3 for *C chinensis*, *S baicalensis*, *P chinense*, and *G jasminoides*. Through a comprehensive network pharmacology approach, a core panel of key active compounds was identified: Kihadalactone A, Phellopterin, Skimmianin, Cavidine, *Obacunone*, and Phellavin, which were predominantly derived from *P chinense*. Kihadalactone A, distinguished by its complex structural scaffold, possesses significant pharmacological value.^[39]^ Phellopterin exerts potent anti-inflammatory effects in the context of atopic dermatitis by effectively inhibiting the activation of STAT3 within keratinocytes.^[40]^ Furthermore, Skimmianin exhibits protective effects against lipopolysaccharide-induced neuroinflammation in microglial cells,^[41]^ and simultaneously inhibits the progression of human esophageal squamous cell carcinoma by suppressing ERK1/2 activation.^[42]^ Cavidine significantly attenuates mechanical allodynia and thermal hyperalgesia in murine models of chemotherapy-induced peripheral neuropathy, while promoting mitophagy to mitigate peripheral nerve injury.^[43]^ *Obacunone* has been shown to possess a broad spectrum of bioactivities, including anti-inflammatory, anti-tumor, anti-obesity, and potent antioxidant properties.^[44]^ It alleviates the deleterious effects of dihydrotestosterone on dermal papilla cells by mitigating apoptosis, cellular senescence, and cell cycle arrest.^[45]^ Furthermore, *Obacunone* orchestrates its multifaceted biological functions through the transcriptional upregulation of Nuclear Factor Erythroid 2-Related Factor 2 and heme oxygenase-1 expression in spinal cord tissues; notably, pharmacological inhibition of the Nrf2 signaling cascade substantially abolishes its analgesic efficacy.^[46]^

Additionally, *Obacunone* suppresses the chronic inflammatory microenvironment in colorectal can,^[47]^ while augmenting astrocytic phagocytic capacity by modulating the CXCR4, NRF2, and ERK1/2 signaling axes, thereby alleviating thalamic pain syndromes.^[48]^ In parallel, elevated MAPK14 expression exacerbates myocardial inflammation.^[49]^ In LPS-induced inflammation, MAPK14 upregulation may be attributed to enhanced protein stability following deubiquitination.^[50]^ In porcine alveolar macrophages, p38 phosphorylation levels are significantly upregulated, triggering the expression of pro-inflammatory mediators, including IL-1α, IL-1β, IL-6, IL-8, and TNF-α.^[51]^ Furthermore, MAPK14 is critical for *N meningitidis* to cross the blood–cerebrospinal fluid barrier and cause meningitis. Specifically, wild-type *N meningitidis* co-opts the MAPK14 pathway in a capsule-dependent manner to facilitate central nervous system invasion.^[52]^ Concurrently, MAPK14 cascade activation amplifies pro-inflammatory gene expression, mediating the inflammatory responses induced by *Glaesserella parasuis*.^[53]^ Ultimately, as a pivotal downstream effector of MyD88, MAPK14 drives neuroinflammation by upregulating IL-1β and IL-33.^[54]^

In the current investigation, we definitively ascertained a pronounced upregulation of MAPK14 protein expression within the established BM cell model. Therapeutic intervention with *Obacunone*, a pharmacologically active monomer derived from the traditional formulation *HLJDT*, substantially attenuated MAPK14 expression, thereby indicating that MAPK14 functions as a cardinal primary drug target. Our comprehensive cellular assays rigorously validated both drug targets and pathogenic molecules at the single-molecule and single-drug levels, providing further corroboration of the predictive validity and guidance of our methodological framework.

Employing a sophisticated network pharmacology-based approach, we successfully characterized *Obacunone* as a critical bioactive constituent. Subsequently, by engineering an in vitro BM simulation utilizing HBMEC, we administered interventions consisting of *Obacunone* and a specific MAPK14 inhibitor, both as distinct monotherapies and in a combinatorial regimen. The empirical data demonstrated that the synergistic combination strategy elicited optimal therapeutic efficacy. This treatment modality significantly potentiated cellular viability, attenuated pathological sodium accumulation across the BBB, mitigated apoptosis, suppressed the biosynthesis of pivotal pro-inflammatory mediators, and reinforced the structural integrity of intercellular tight junctions.

The present study is subject to certain limitations that warrant acknowledgment. Primarily, the research relies heavily on an in vitro Transwell co-culture system. Although this model is capable of partially simulating the permeability and inflammatory responses of the BBB, it fails to fully recapitulate the complex physiological microenvironment present in vivo. In the in vivo setting, the BBB functions as an integral component of the neurovascular unit, which comprises a complex ecosystem of endothelial cells, pericytes, astrocytic end-feet, and the basement membrane. Furthermore, the absence of direct morphological assessments implies that relying solely on quantitative protein evidence derived from Western blot analysis renders the findings somewhat 1-sided.

In conclusion, this study employed network pharmacology and bioinformatics to elucidate the main active components, core targets, and signaling pathways of *HLJDT* in treating BM. It further corroborates that *HLJDT* constitutes an effective multicomponent, multi-target, and multi-pathway therapeutic modality for BM, providing a robust foundation for its future drug development and clinical application. Molecular docking experiments validated the binding affinity of *Obacunone* and MAPK14, and various experimental methodologies confirmed the therapeutic efficacy of *Obacunone* and the MAPK14 inhibitor in cellular models of BM.

## Author contributions

**Conceptualization:** Yan Xiao, Xiuqing Tong.

**Formal analysis:** Yan Xiao.

**Methodology:** Yan Xiao.

**Writing – original draft:** Yan Xiao.

**Writing – review & editing:** Xiuqing Tong.


